# Alternative Splicing Events Identified in Human Embryonic Stem Cells and Neural Progenitors

**DOI:** 10.1371/journal.pcbi.0030196

**Published:** 2007-10-26

**Authors:** Gene W Yeo, Xiangdong Xu, Tiffany Y Liang, Alysson R Muotri, Christian T Carson, Nicole G Coufal, Fred H Gage

**Affiliations:** 1 Crick-Jacobs Center for Theoretical and Computational Biology, Salk Institute, La Jolla, California, United States of America; 2 Laboratory of Genetics, Salk Institute, La Jolla, California, United States of America; University College London, United Kingdom

## Abstract

Human embryonic stem cells (hESCs) and neural progenitor (NP) cells are excellent models for recapitulating early neuronal development in vitro, and are key to establishing strategies for the treatment of degenerative disorders. While much effort had been undertaken to analyze transcriptional and epigenetic differences during the transition of hESC to NP, very little work has been performed to understand post-transcriptional changes during neuronal differentiation. Alternative RNA splicing (AS), a major form of post-transcriptional gene regulation, is important in mammalian development and neuronal function. Human ESC, hESC-derived NP, and human central nervous system stem cells were compared using Affymetrix exon arrays. We introduced an outlier detection approach, REAP (Regression-based Exon Array Protocol), to identify 1,737 internal exons that are predicted to undergo AS in NP compared to hESC. Experimental validation of REAP-predicted AS events indicated a threshold-dependent sensitivity ranging from 56% to 69%, at a specificity of 77% to 96%. REAP predictions significantly overlapped sets of alternative events identified using expressed sequence tags and evolutionarily conserved AS events. Our results also reveal that focusing on differentially expressed genes between hESC and NP will overlook 14% of potential AS genes. In addition, we found that REAP predictions are enriched in genes encoding serine/threonine kinase and helicase activities. An example is a REAP-predicted alternative exon in the *SLK* (serine/threonine kinase 2) gene that is differentially included in hESC, but skipped in NP as well as in other differentiated tissues. Lastly, comparative sequence analysis revealed conserved intronic *cis*-regulatory elements such as the *FOX1/2* binding site GCAUG as being proximal to candidate AS exons, suggesting that *FOX1/2* may participate in the regulation of AS in NP and hESC. In summary, a new methodology for exon array analysis was introduced, leading to new insights into the complexity of AS in human embryonic stem cells and their transition to neural stem cells.

## Author Summary

Deriving neural progenitors (NP) from human embryonic stem cells (hESC) is the first step in creating homogeneous populations of cells that will differentiate into myriad neuronal subtypes necessary to form a human brain. During alternative RNA splicing (AS), noncoding sequences (introns) in a pre-mRNA are differentially removed in different cell types and tissues, and the remaining sequences (exons) are joined to form multiple forms of mature RNA, playing an important role in cellular diversity. The authors utilized Affymetrix exon arrays with probes targeting hundreds of thousands of exons to study AS comparing human ES to NP. To accomplish this, a novel computational method, REAP (Regression-based Exon Array Protocol), is introduced to analyze the exon array data. The authors showed that REAP candidates are consistent with other types of methods for discovering alternative exons. In addition, REAP candidate alternative exons are enriched in genes encoding serine/theronine kinases and helicase activities. An example is the alternative exon in the *SLK* (serine/threonine kinase 2) gene that is included in hESC, but excluded in NP as well as in other differentiated tissues. Finally, by comparing genomic sequences across multiple mammals, the authors identified dozens of conserved candidate binding sites that were enriched proximal to REAP candidate exons.

## Introduction

The human central nervous system is composed of thousands of neuronal subtypes originating from neural stem cells (NSCs) that migrate from the developing neural tube. Such neuronal complexity is generated by a vast repertoire of molecular, genetic, and epigenetic mechanisms, such as the active retrotransposition of transposable elements [1], alternative promoter usage, alternative RNA splicing (AS), alternative polyadenylation, RNA editing, post-translational modifications, and epigenetic modulation [2]. Understanding the processes that generate neuronal diversity is key to gaining insights into neuronal development and paving new avenues for biomedical research.

Human embryonic stem cells (hESCs) are pluripotent cells that propagate perpetually in culture as undifferentiated cells and can be induced to differentiate into a multitude of cell types both in vitro and in vivo [3]. As hESCs can theoretically generate all cell types that make up an organism, they serve as an important model for understanding early human embryonic development. In addition, the hESCs are a nearly infinite source for generating specialized cells such as neurons and glia for potential therapeutic purposes [4,5]. In recent years, methods have been introduced to induce hESCs to differentiate into neural progenitors (NPs) [6,7] and neuronal and glial subtypes [8–12]. The therapeutic interest in understanding the molecular basis of pluripotency and differentiation has led to many studies comparing transcriptional profiles in different hESC lines and the study of expression changes during the differentiation of hESCs to various lineages [13–17].

NSCs and progenitor cells (NPs) are present throughout development and persist into adulthood [18–20]. They are critical for both basic research and developing approaches to treat neurological disorders, such as Parkinson disease and amyotrophic lateral sclerosis (ALS), and stroke or head injuries [21,22]. NSCs and NPCs can be isolated from human fetal brain tissue [23–26], as well as from several regions of the adult human brain, such as the cortex, hippocampus, and the subventricular zone (SVZ) of the lateral ventricles [26–35]. Several studies have explored expression patterns of NPCs. For example, Wright et al. identified “expressed” and “not expressed” genes in NPCs isolated from the human embryonic cortex [24]; Cai et al. used the massively parallel signature sequencing profiling (MPSS) technique to analyze expression of fetal NPCs in comparison to hESCs and astrocyte precursors [27]; Maisel et al. used Affymetrix Gene Chip arrays to compare adult and fetal NPCs propagated in neurospheres [35]. However, as with hESCs, the focus thus far has been primarily on transcriptional differences, which ignores differential RNA processing such as AS, polyadenylation, degradation, or promoter usage.

AS is frequently used to regulate gene expression and to generate tissue-specific mRNA and protein isoforms [36–39]. Recent studies using splicing-sensitive microarrays suggested that up to 75% of human genes undergo AS, where multiple isoforms are derived from the same genetic loci [40]. This functional complexity underscores the challenge and importance of elucidating AS regulation. AS appears to play a dominant role in regulating neuronal gene expression and function [41,42]. Examples of splicing regulators that are enriched and function specifically in neuronal cells include the brain-specific splicing factor *Nova* [43,44] and neural-specific polypyrimidine tract binding protein (*nPTB*), which antagonizes its paralogous *PTB* to regulate exon exclusion in neuronal cells [45–47]. Finally, an early report estimating that 15% of point mutations disrupt splicing underscores the importance of splicing in human disease [48]. Indeed, the disruption of specific AS events has been implicated in several human genetic diseases, such as frontotemporal dementia and parkinsonism, Frasier syndrome, and atypical cystic fibrosis [49]. While insights into the regulation of AS have come predominantly from the molecular dissection of individual genes [36,49], it is becoming clear that molecular rules can be identified from large-scale studies of both constitutive splicing and AS [40].

Most systematic global analyses on AS have focused on comparisons across differentiated human tissues [50–52]. Only one study, utilizing expressed sequence tag (EST) collections from stem cells, has attempted to find AS differences between embryonic and hematopoietic stem cells [53]. However, utilizing ESTs to identify AS has intrinsic problems, as ESTs tend to be biased for the 3′ ends of genes, and full coverage of the genome by ESTs is severely limited by sequencing costs. The commercial availability of Affymetrix exon arrays provides an alternative approach to interrogate the expression of every known and predicted exon in the human genome. The Affymetrix GeneChip Human Exon 1.0 ST array contains ∼5.4 million features used to interrogate ∼1 million exon clusters (collections of overlapping) of known and predicted exons with more than 1.4 million probesets, with an average of four probes per exon.

Our goal was to identify and characterize AS events that distinguish pluripotent hESCs from multipotent NPs, paving the way for future candidate gene approaches to study the impact of AS in hESCs and NPs. However, as different hESC lines were established under different culture conditions from embryos with unique genetic backgrounds, we expected that hESCs and their derived NPs might have distinct epigenetic and molecular signatures [54]. As both common and cell-line specific alternatively spliced exons are likely to be important in regenerative research, in our study two separate hESC lines were used, with independent protocols for differentiating the hESCs into NPs positive for *Sox1*, an early neuroectodermal marker. As an endogenously occurring population of NPs, human central nervous system stem cells grown as neurospheres (hCNS-SCns) were utilized as a natural benchmark for derived NPs. We developed an approach called REAP (Regression-based Exon Array Protocol), which is based on robust regression that analyzed signal estimates from Affymetrix exon array data to identify AS exons. Experimental validation revealed alternative exons that distinguish hESCs from NPs; some of them also distinguish hESCs from a variety of differentiated tissues. A comparison of REAP-predicted alternative events with independent methods, such as using publicly available transcripts (ESTs and mRNAs) and computational predictions based on genomic sequence information alone [55], showed a strong concordance of REAP-identified AS exons with AS events identified from these orthogonal methods. Finally, using analysis of the sequences flanking REAP-identified alternative exons, we were able to discover known and novel *cis-*regulatory elements that potentially regulate these AS events.

## Results

### Derivation of Neural Progenitors from Embryonic Stem Cells

**Figure 1:**
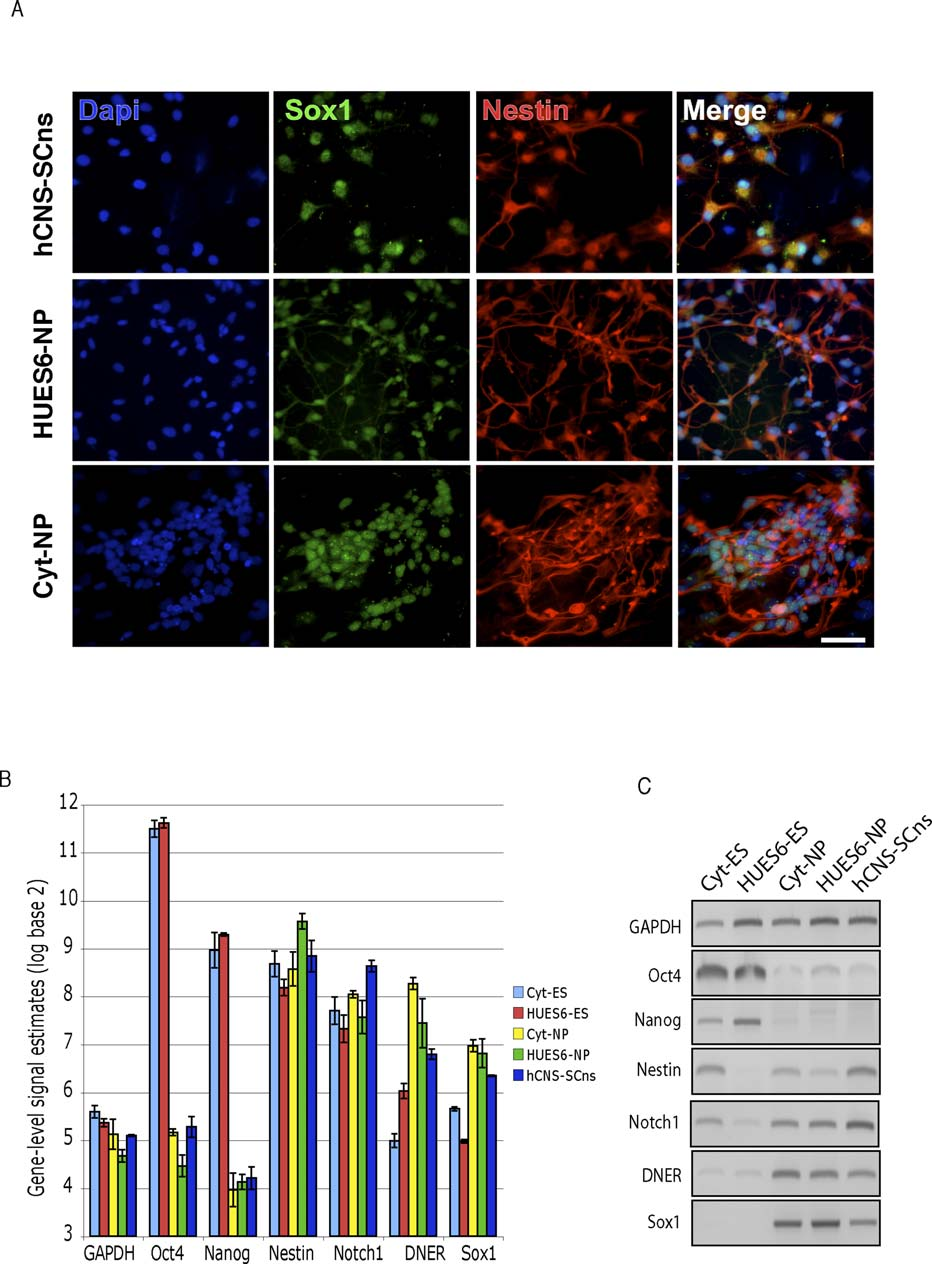
Molecular Characterization of Human Embryonic Stem Cell Lines and Neuronal Progenitors (A) Immunohistochemical analysis of markers in NPs derived from the hESC lines (Cyt-NP from Cyt-ES; and HUES6-NP from HUES6-ES) and in hCNS-SCns. Cyt-NP, HUES6-NP, and hCNS-SCns cells were *Nestin* and *Sox1* positive. Nuclei stained positive for Dapi. White horizontal bar indicated 15 μm. (B) Gene-level signal estimates of marker genes (*GAPDH*, *Oct4*, *Nanog*, *Nestin*, *Notch1*, *DNER*, and *Sox1*) from Affymetrix exon array analysis. Vertical bars indicated average log_2_ normalized signal estimates, and error bars represented standard deviations from three independent replicate experiments per cell type. (C) RT-PCR of marker genes (*GAPDH*, *Oct4*, *Nanog*, *Nestin*, *Notch1*, *DNER*, and *Sox1*).

NPs were independently derived from two hESC lines, and RNA extracted from the cell lines was processed and hybridized onto Affymetrix Human 1.0 ST exon arrays. Immunohistochemical and reverse-transcriptase polymerase chain reaction (RT-PCR) analyses demonstrated that the hESCs expressed pluripotent marker genes, and the derived NPs expressed multipotent and neurogenic markers similar to hCNS-SCns. Undifferentiated Cythera (Cyt-ES) and HUES6 (HUES6-ES) hESC lines were maintained in culture as previously described [12,23,56]. Utilizing specific antibodies, we observed that undifferentiated Cyt-ES and HUES6-ES cells were positive for the pluripotent markers *Oct4*, *SSEA-4*, and T*ra-1–80* (unpublished data). NPs were derived from the hESC cell lines using protocols optimized for each line (see Materials and Methods). Greater than 90% of derived NP cells (Cyt-NP from Cyt-ES and HUES6-NP from HUES6-ES) were positive for *Sox1*, an early neuroectodermal marker, and *Nestin* (Figure 1A), and negative for Oct4 (unpublished data). As a natural benchmark for the derived NPs, we utilized hCNS-SCns, which were previously isolated from fresh human fetal brain tissues using antibodies to cell-surface markers and fluorescence-activated cell sorting [12,23]. The hCNS-SCns form neurospheres in culture which are greater than 90% *Nestin* and *Sox1* positive, and differentiate into both neurons and glial cells in vitro [12,23]. Immunohistochemical analysis confirmed that hCNS-SCns were negative for Oct4 (unpublished data) and positive for *Sox1* and *Nestin* (Figure 1A).

Here, known molecular markers were subjected to RT-PCR measurements, which were compared to gene-level signal estimates generated from the exon array data. Total RNA was extracted, and labeled cDNA targets were generated from three independent preparations of each cell type, namely Cyt-ES, HUES6-ES, Cyt-NP, HUES6-NP, and hCNS-SCns. To facilitate downstream analyses, instead of utilizing the meta-gene sets available from the manufacturers, we generated our own gene models by clustering alignments of ESTs and mRNAs to annotated known genes from the University of California Santa Cruz (UCSC) Genome Browser Database. After hybridization, scanning, and extraction of signal estimates for each probeset on the exon arrays, gene-level estimates were computed based on our gene models using available normalization and signal estimation software from Affymetrix. For every gene, a *t*-statistic and corresponding *p*-value were computed representing the relative enrichment of the expression of the gene in hESC versus NP, such as in Cyt-ES versus Cyt-NP. After correcting for multiple hypothesis testing using the Benjamini-Hochberg method, a *p*-value cutoff of 0.01 was used to identify enriched genes. Close inspection of all pairs of hESC-NP comparisons revealed a generally significant overlap from 31% to 85% of the smaller of two compared sets of enriched genes (see Figure S1). Thus for the purpose of identifying overall pluripotent and neural lineage-specific genes, the collective set of NPs (Cyt-NP, HUES6-NP, and hCNS-SCns) was compared to the collective set of hESCs (Cyt-ES and HUES6-ES).

*Oct4* and *Nanog*, which are important in maintaining the pluripotent state of embryonic stem cells (ESCs), were highly expressed in hESCs but were significantly lower in NPs (Figure 1B). RT-PCR of *Oct4* and *Nanog* mRNA levels accurately reflected the signal estimates from the array (Figure 1C). Interestingly, *Nestin* was not significantly higher in NPs as compared to the hESC from the gene-level estimates (*p*-value 0.065), which was further confirmed by RT-PCR (Figure 1C). *Notch* was recently identified to be important in promoting the neural lineage entry in mouse ESCs [57] and was shown to regulate stem cell proliferation in somatic mouse and hESC [58]. Gene-level signal estimates suggested that *Notch* was significantly higher in hCNS-SCns relative to hESCs, but levels of *Notch* were not significantly different in the derived NPs compared to hESCs. Delta/Notch-like EGF-related receptor (*DNER)*, a neuron-specific transmembrane protein, was recently shown to bind to *Notch* at cell–cell contacts and activates *Notch* signaling in vitro [59]. RT-PCR validation of *DNER* confirmed array-derived signal estimates, indicating an enrichment of *DNER* in NPs relative to hESCs (Figure 1C). Finally, *Sox1*, a HMG-box protein related to SRY, was shown to be one of the earliest transcription factors expressed in cells committed to the neural fate [60]. Here the gene-level estimates indicated that *Sox1* was expressed significantly higher in NPs relative to hESCs (*p*-value 0.00013, Figure 1B), a finding that was confirmed by RT-PCR (Figure 1C).

From these examples, we concluded that RT-PCR validation correlated well with gene-level estimates from the exon array. In addition, the derived NPs had decreased levels of pluripotent markers *Oct4* and *Nanog* but had levels of *Sox1* that were comparable to hCNS-SCns. This finding confirmed that the derived NPs were committed to a neural fate and validated the use of hCNS-SCns as a benchmark for NPs.

Next we asked whether the highest enriched genes in hESCs relative to NPs reflected our existing knowledge in the literature. Using the above-mentioned groupings of hESCs (Cyt-ES, HUES6-ES) and NPs (Cyt-NP, HUES6-NP, and hCNS-SCns), 2,945 genes were enriched in hESCs relative to NPs; and 552 genes were enriched in the NPs relative to hESCs, at a *p*-value significance cutoff of 0.01 (correcting for multiple hypothesis testing using the Benjamini-Hochberg method). The 15 most enriched genes in hESCs included genes such as teratocarcinoma-derived growth factor 1 (*TDGF1/cripto; p-*value < 10^−12^), zinc finger protein 42 (*Zfp42/Rex1*; *p-*value < 10^−12^), *Oct4* (*p-*value < 10^−12^), *Nanog* (*p-*value < 10^−10^), *lin-28 homolog* (*p-*value < 10^−10^), *cadherin 1 preprotein* (*p-*value < 10^−10^), *claudin 6* (*p-*value < 10^−9^), *ephrin receptor EphA1* (*p-*value < 10^−9^), and *erbB3* (*p-*value < 10^−9^). *TDGF1/cripto* was first shown to stimulate DNA synthesis and cell proliferation of both undifferentiated and differentiated embryonic carcinoma cells [61] and was later shown to be important for cardiomyocyte formation from mouse ESC [62]. *Oct4*, reviewed in [63], and *Nanog* [64] are crucial for the pluripotency of hESCs. Recently, knockdown of *Zfp42/Rex-1* in mouse ESC caused the cells to differentiate [65]. Our gene-level exon array analysis confirmed that the hESCs and NPs were molecularly distinct.

**Figure 2:**
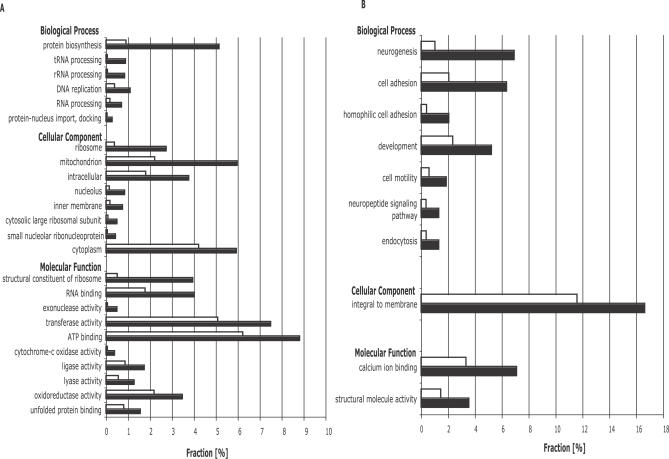
Gene Ontology Analysis Differential gene expression of hESCs (Cyt-ES and HUES6-ES) and NPs (Cyt-NP, HUES6-NP, and hCNS-SCns) was computed from gene-level signal estimates. Statistical significance for differential gene expression was determined by using *t*-statistics with Benjamini-Hochberg correction for false discovery rate (*p <* 0.01). Gene Ontology “molecular function,” “cellular component,” and “biological process” categories, which differed significantly (*p* < 0.05) in the representation between significantly enriched genes (black bars) and all other genes (white bars), were shown. Statistical significance for GO analysis was assessed by using χ^2^ statistics with Bonferroni correction for multiple hypothesis testing. GO categories are ordered from top to bottom in order of decreasingly significant bias toward enriched genes. (A) GO analysis of enriched genes in hESCs. (B) GO analysis of enriched genes in NPs.

To reveal global functional differences between the enriched genes in hESCs or NPs, the enriched genes were subjected to a Gene Ontology (GO, http://www.geneontology.org) analysis as described previously [55]. Enriched genes in hESCs were more likely to be in molecular function categories, such as “RNA binding” (*p-*value < 10^−12^), “structural constituent of ribosome” (*p-*value < 10^−51^), “exonuclease activity” (*p-*value < 10^−6^), “cytochrome-c oxidase activity” (*p-*value < 10^−5^), and “ATP binding” (*p-*value < 10^−6^), and in biological processes involved with “tRNA processing” (*p-*value < 10^−6^) and “protein biosynthesis” (*p-*value < 10^−48^), consistent with our knowledge of hESCs as a rapidly proliferating population of cells (Figure 2A). Similar analysis of the enriched genes in NPs revealed an overrepresentation in molecular functional categories, such as “calcium ion binding” (*p-*value < 10^−8^) and “structural molecule activity” (*p-*value < 10^−5^), and in biological processes involved with “neurogenesis” (*p-*value < 10^−38^), “cell adhesion” (*p-*value < 10^−13^), “cell motility” (*p-*value < 10^−4^), “development” (*p-*value < 10^−6^), “neuropeptide signaling pathway” (*p-*value < 10^−4^), and “endocytosis” (*p-*value < 10^−4^) (Figure 2B). Considering that these were the only categories that were significantly enriched out of more than 18,000 GO terms, and that randomly selected sets of similar numbers of genes did not reveal statistical differences in GO categories, our results confirmed that the global molecular profiles derived from exon array analysis were consistent with known differences between hESCs and NPs.

To summarize, firstly immunohistochemical and RT-PCR evidence validated that the cells exhibited expected characteristics; secondly, stage-specific marker gene differences by RT-PCR were reflected accurately by gene-level estimates from the exon arrays; thirdly, the hESC-enriched genes were coherent with known genes that controlled pluripotency and self-renewal; and lastly, the global functional profiles exemplified expected biological differences between hESC and NP cells.

### Description of the Regression-Based Exon Array Protocol

Convinced that the signal estimates from the exon arrays reflected expected molecular and biological differences between hESCs and NPs, we sought to identify AS events. We compared Cyt-ES to hCNS-SCns to illustrate our approach. First we normalized the data and generated signal estimates with Robust Multichip Analysis (RMA) and estimated the probability that each probeset was detected above background (DABG) using publicly available Affymetrix Power Tools (APT). We analyzed probesets that (i) comprised three or more individual probes; (ii) were localized within the exons of our gene models with evidence from at least three sources (mRNA, EST, or full-length cDNA); and (iii) were detected above background in at least one of the cell lines. In total, 17,430 gene models were represented by probesets that satisfied these criteria.

**Figure 3:**
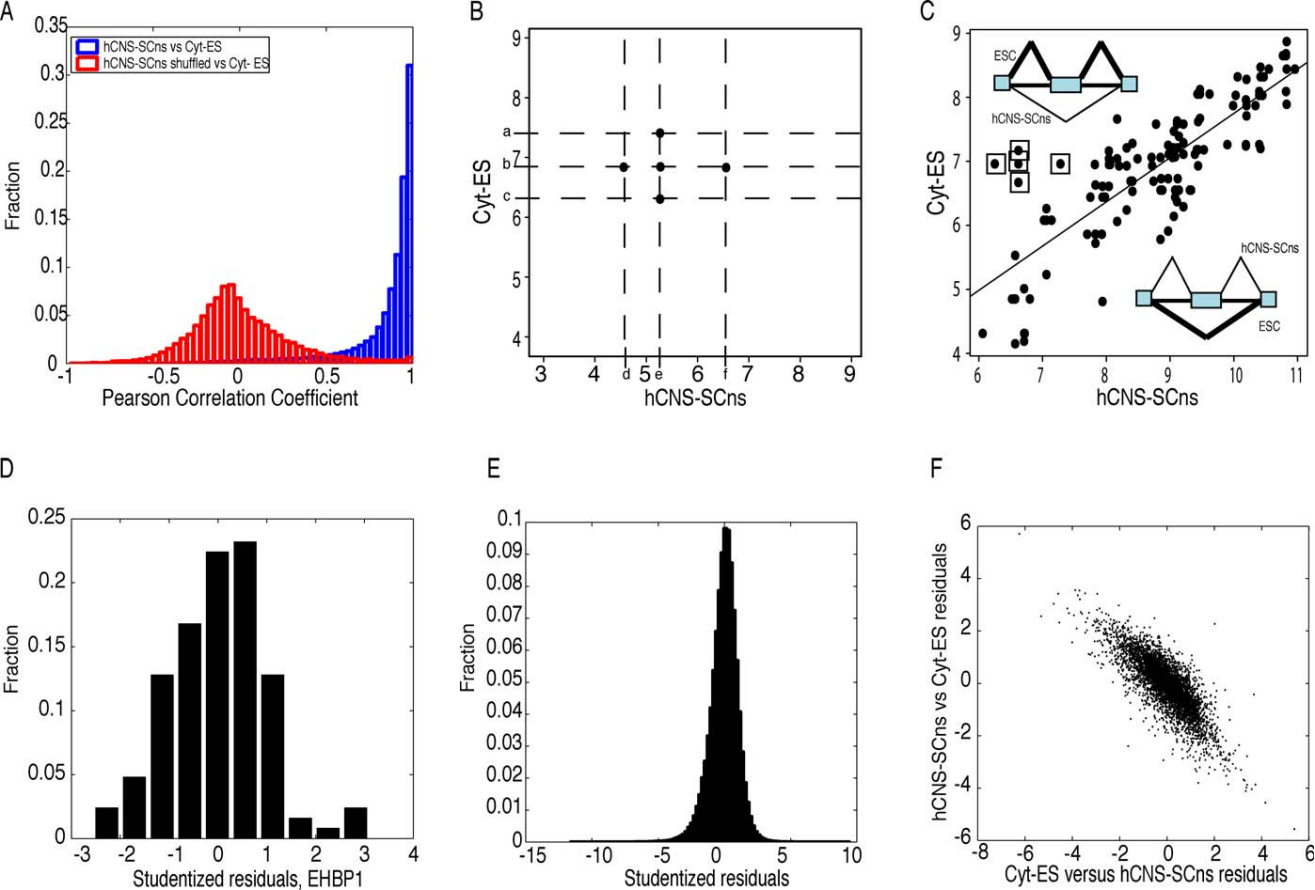
Description of the REAP Algorithm Comparing Exon Array Signal Estimates from hCNS-SCns and Cyt-ES (A) Histogram of Pearson correlation coefficients computed from median signal estimates for probesets between Cyt-ES versus hCNS-SCns for genes (blue bars). Genes were required to have more than five probesets localized within the exons in the gene. Red bars represented Pearson correlation coefficients computed from exons with shuffled signal estimates. (B) Each probeset contained probeset-level estimates from three replicates each, (a, b, c) in Cyt-ES and (d, e, f) in hCNS-SCns. The five points summarizing the log_2_ probeset-level estimates are indicated by black filled circles. (C) Each probeset was summarized by five points. Scatter plots of signal estimates for probesets that were present in at least one cell type (Cyt-ES or hCNS-SCns) for the *EHBP1* gene. Probesets were considered present if the DABG p-value was <0.05 for all three replicates in the cell type. A regression line derived from robust linear regression with MM estimation is indicated. Points above the line represent probesets within exons that were enriched in Cyt-ES and points below represent exons that were enriched in hCNS-SCns. Points close to the regression line are not significantly different in Cyt-ES versus hCNS-SCns. Boxed points represented the five-point summary of a probeset that was significantly enriched in Cyt-ES but was skipped in hCNS-SCns. (D) Histogram of studentized residuals for points from the scatter plot in (C) in *EHBP1*. (E) The histogram of studentized residuals for all points for all analyzed probesets (100 bins). (F) The scatter plot of studentized residuals generated from comparing Cyt-ES versus hCNS-SCns and hCNS-SCns versus Cyt-ES of 5,000 randomly chosen probesets.

Next we asked whether the probeset expression within each gene model was positively correlated for any two cell lines. To do this we calculated the Pearson correlation coefficient between the vectors of median signal estimates across replicates in Cyt-ES versus hCNS-SCns. The vast majority of genes (>80%) was found to have probeset-level Pearson correlation coefficients of greater than 0.8 (Figure 3A). Next we randomly permuted the association between the median signal estimates and the probesets for each gene in hESCs (or hCNS-SCns) and observed that the distribution of Pearson correlation coefficients for the permuted sets was centered at zero, as expected (Figure 3A). This indicated that the signal estimates for probesets between hESCs and hCNS-SCns were highly correlated and suggested that a scatter plot of probeset signal estimates between hESCs and hCNS-SCns would reveal a linear relationship for the majority of genes. We hypothesized that a linear regression to determine if some probesets behaved unexpectedly in one cell type compared to the other might be a reasonable approach to identify AS exons.

Here, a possible representation of the data was explored. If we had N replicates in one condition and M replicates in the other, we could consider N*M points if we analyzed every possible pairing. For instance, three replicate signal estimates for every probeset per cell line, such as signal estimates a, b, and c in hESCs and d, e, and f in hCNS-SCns, would translate to pairing every signal (d,a), (d,b), (d,c) … (f,a), (f,b), (f,c) for linear regression (Figure 3B). Instead, pairing the signal estimates of all replicates in one condition to the median of the other would only require N + M − 1 points and would capture the variation of the signal estimates of each probeset. For example, we considered (d,b), (e,a), (e,b), (e,c), and (f,b) points where b and d were the median intensities for the replicates in Cyt-ES and hCNS-SCns, respectively (Figure 3B). A scatter plot of all probesets of the *EHBP1* (EH domain binding protein, RefSeq identifier NM_015252) is shown in Figure 3C in the format described. Each probeset was represented by 5 points of log-transformed (base 2) values; and each point on the scatter plot reflected the extent of inclusion of an exon in hESCs and in hCNS-SCns (Figure 3C).

A classical linear regression model could be proposed to fit the response variable y_ij_, the log_2_ expression of probeset i in cell-type j (for example, j is Cyt-ESC) to explanatory variables x_ik_, and the log_2_ expression of probeset i in cell type k (for example, k is hCNS-SCns). However, classical linear regression by least-squares estimation is biased because the least squares predictions are strongly influenced by the outliers, leading to completely incorrect regression line estimates, masking of the outliers, and incorrect predictions of outliers. Therefore, we applied M-estimation robust regression to estimate the line, which is less sensitive to outliers. Fitting was performed using an iterated, re-weighted least squares analysis. Our assumption was that most of the points were “correct,” i.e., that most of the exons were constitutively spliced. Thus, robust regression would find the line that was least dependent on outliers, which would be potential AS exons. This assumption was substantiated by our observation that, using publicly available ESTs and mRNAs, a minority of human exons (7%) have evidence for exon-skipping, the most common form of AS. Using robust regression, the regression line for Cyt-ESC versus hCNS-SCns in the *EHBP1* gene is illustrated in Figure 3C. The boxed points belonged to a probeset that was enriched in hESCs but depleted in hCNS-SCns, which was suspected to be due to AS. The difference between the actual and regression-based predicted value, normalized by the estimate of its standard deviation, is called the studentized residuals. Studentized residuals were computed for all probeset pairs in *EHBP1*, and the histogram depicting their distribution is illustrated in Figure 3D. As expected, the mean of the distribution was close to zero, and the distribution was approximated by a *t*-distribution with n-p-1 degrees of freedom, where n was the number of points on the scatter plot, and the number of parameters p was 2. The boxed points had studentized residuals of 1.829, 3.104, 2.634, 3.012, and 2.125 with *p*-values of 0.034, 0.00119, 0.00477, 0.00158, and 0.01780, respectively, computed based on the *t*-distribution (Figure 3C). At a stringent *p*-value cutoff of 0.01, four of the five studentized residuals were designated as significant “outliers,” indicating that the probeset was “unusual.” RT-PCR confirmed that the exon, represented by the probeset, was indeed differentially included in hESCs and skipped in hCNS-SCns (Figure 7B). Applying this approach to all gene models revealed that, as expected, the majority of studentized residuals are centered at zero (Figure 3E). Thus far in the example, our analysis was based on regression of hESCs (*y*-axis) versus hCNS-SCns (*x*-axis) (Figure 3B–3D). However, robust regression as described was not symmetrical, i.e., parameter estimation of y as a function of x was not the same as that of x as a function of y. The negative slope revealed that probesets enriched in hESCs versus hCNS-SCns (positive valued), were expectedly depleted when hCNS-SCns was compared to hESCs (negative valued; Figure 3F). As our method for predicting candidate alternative exons was based on identification of outliers using robust regression, we named the method REAP.

### Identification and Removal of False Positives

**Figure 4:**
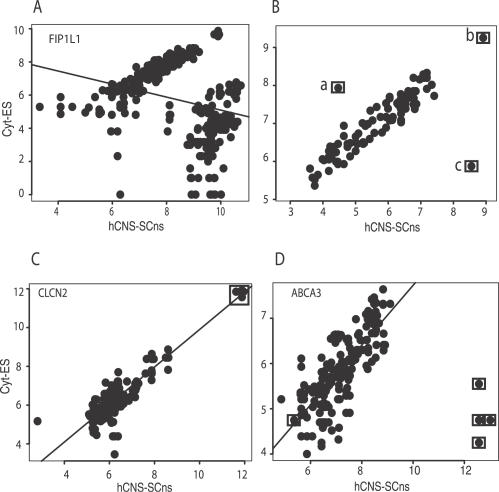
Sources of False Positives (A) Scatter plot of points for the *FIP1L1* gene and the line representing the robust regression estimate. (B) Boxed point “a” represents a significant “outlier” (with a significantly different studentized residual and low leverage). Boxed point “b” represents a “high leverage” point (low studentized residual and a high leverage). Boxed point “c” represents a “high influence” point (high studentized residual, high leverage, and high influence). (C) Scatter plot of points for the *CLCN2* gene. Boxed points represent “high leverage” points. (D) Scatter plot of points for the *ABCA3* gene. Boxed points represent “high influence” points.

In the process of experimentally validating our predictions, we encountered three main sources of false positives (FP) from robust regression. First, we identified genes with probeset signal estimates that were poorly correlated and were not amenable to our method. As an example, the median probeset signal estimates in hESCs and hCNS-SCns of the *FIP1L1* gene (gene identifiers BC011543, AL136910) had a Pearson correlation coefficient of 0.38, and the distribution of points was not amenable to robust regression (Figure 4A). To avoid inappropriate application of REAP and generating false predictions, we empirically determined that a gene had to have a Pearson correlation coefficient cutoff of 0.6 before being amenable to REAP analysis. Next, we managed two additional sources of FPs, namely “high-leverage” and “high-influence” points, which we were able to identify by computing the following metrics. For every point, we computed (i) the studentized residual (as described above), (ii) the influence, and (iii) the leverage (see Materials and Methods for more details). Leverage assessed how far away a value of the independent variable was from the mean value; the farther away the observation the more leverage it had. The influence of a point was related to its covariance ratio: a covariance ratio larger (or smaller) than 1 implied that the point was closer (or farther) than was typical to the regression line, so removing it would hurt (or help) the accuracy of the line and would increase (or decrease) the error term variance. Influence was computed as the absolute difference between the covariance ratio and unity. To illustrate further, a point was classified as an “outlier” if it had a large studentized residual (*p* < 0.01) and low leverage (boxed point “a”); as a “high-leverage” point if it had a low studentized residual and high leverage (boxed point “b”); and as a “high-influence” point if it had a high studentized residual, high leverage, and high influence (boxed point “c”; Figure 4B). Points that resembled boxed point “a” were designated as potential AS events. For example, four of the five boxed points in Figure 3C were “outliers,” and RT-PCR validation indicated that the exon represented by the probeset was indeed skipped in hCNS-SCns (*EHBP1*, Figure 7B). Points that were “high-leverage,” such as the five points in the *CLCN2* gene, were experimentally verified to be a FP (Figure 4C; unpublished data). Points that were “high-influence,” such as the four of five boxed points in the *ABCA3* gene were also experimentally verified to be a FP (Figure 4D*;* unpublished data). In conclusion, in order to reduce the FP rate, all points were evaluated according to the metrics described, and points that were significant “outliers” were considered putative AS events.

### Global Identification and Characterization of REAP[+] Exons

REAP was applied to identify AS events in NPs compared to hESCs: Cyt-NP versus Cyt-ES; HUES6-NP versus HUES6-ES; hCNS-SCns versus Cyt-ES, and hCNS-SCns versus HUES6-ES. After removing potential FPs, 11,348 genes containing 158,657 probesets were scored by REAP.

**Figure 5:**
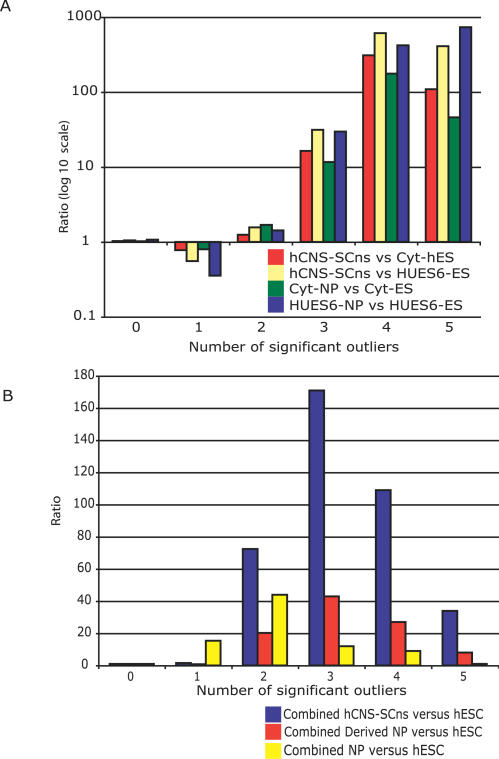
Correlation between “Outliers” (A) The number of probesets with N significant “outliers” was determined for hCNS-SCns versus Cyt-ES, hCNS-SCns versus HUES6-ES, Cyt-NPs versus Cyt-ES, and HUES6-NPs versus HUES6-ES (N = 0, 1, 2, 3, 4, 5). For comparison, points to probeset relationships were randomly permuted, retaining the same number of “outliers.” Vertical bars represent the ratio between the number of actual points and the randomly permutated sets. (B) Similar to (A), except points were counted as “outliers” only if they were “outliers” in both hCNS-SCns versus Cyt-ES and hCNS-SCns versus HUES6-ES (combined hCNS-SCns versus hESC; blue bars); in both HUES6-NP versus HUES6-ES and Cyt-NP versus Cyt-ES (combined derived NP versus hESC; red bars); and in all four comparisons (combined NP versus hESC; yellow bar).

As described above, for each pair of cell lines compared, each probset was represented by five points, where each point was defined a significant outlier if it had a high residual (*p* < 0.01), low influence, and high leverage. Points per probeset should be correlated; in other words, if one point was a significant outlier, the other points were expected to be outliers as well. To ensure that this was the case, we counted the number of probesets with N significant outliers, where N was varied from 0 to 5. Next, the identity of the probesets and points derived from them were exchanged with other probesets, keeping constant the total number of points that were considered significant outliers. At N = 0, we observed approximately equal numbers of probesets in the actual versus shuffled controls. In contrast, we observed that there were 1.5 times more probesets with N = 2 significant outliers relative to shuffled controls; 12–31 times more probesets with N = 3; and 17–612 times more probesets that had N = 4 significant outliers (Figure 5A; see Table S1). For example, in hCNS-SCns compared to Cyt-ES, approximately 0.39% (490 of 124,604) of probesets had three significant outliers and 0.25% (308 probesets) had four significant outliers, relative to 0.02% and 0% of shuffled controls, respectively.

Next we asked whether the overlap between related comparisons was higher than expected. Comparing the significant probesets between hCNS-SCns versus Cyt-ES and hCNS-SCns versus HUES6-ES revealed 672 significant probesets (N ≥ 2), whereas if we shuffled the associations between probeset identity and significant outliers, only four significant probesets (N ≥ 2) were identified—a 168-fold enrichment (Figure 5B, Table S1). A total of 236 significant probesets overlapped when we compared the derived NPs to hESCs (Cyt-NP versus Cyt-ES and HUES6-NP versus HUES6-ES), relative to seven significant probesets (34-fold enrichment).

At a cutoff of two significant outliers, 1,737 probesets contained in internal exons were defined as positive REAP predictions (hereafter called REAP[+]) exons—candidate AS events that distinguished NP from hESC. Surprisingly, we observed that the majority of REAP[+] exons were specific to the pair of hESC and NP that was compared, likely reflecting differences in genetic origins and/or culturing and differentiation conditions of the cell lines: 614 REAP[+] events were unique to hCNS-SCns versus HUE6-ES; 220 were unique to hCNS-SCns versus Cyt-ES; 439 were unique to HUES6-NP versus HUES6-ES; and 250 were unique to Cyt-NP versus Cyt-ES. The shared events between pairs of comparisons made up a minority of the total number identified: 102 REAP[+] events were found to be in common between hCNS-SCns versus Cyt-ES and hCNS-SCns versus HUES6-ES; 48 between hCNS-SCns versus HUES6-ES and HUES6-NP versus HUES6-ES; and only 17 between hCNS-SCns versus Cyt-ES and Cyt-NP versus Cyt-ES (Table S2).

### Comparison of REAP to EST-Based Method and ACEScan

**Figure 6:**
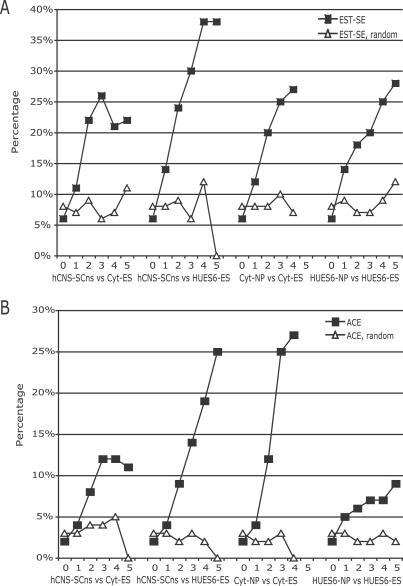
Comparison of REAP Predictions for hCNS-SCns versus Cyt-hES, hCNS-SCns versus HUES6-ES, Cyt-NP versus Cyt-ES, and HUES6-NPs versus HUES6-ES with Alternative Exons Identified by an EST-Based Method and ACEScan (A) Black-filled squares represented the fraction of exons containing probesets with N significant points that had EST evidence for exon inclusion or exclusion (N = 0, 1, 2, 3, 4 and 5). White-filled triangles represented similarly computed fractions with permuted probeset to exon mappings. (B) Black-filled squares represented the fraction of exons containing probesets with N significant points that had ACEScan positive scores, indicative of evolutionarily conserved alternative exons. White-filled triangles represented similarly computed fractions with permuted probeset to exon mappings.

Traditionally, AS exons were discovered by using EST alignments to genomic loci, and also more recently by computational algorithms that used sequence information extracted from multiple genomes. Here, we compared REAP predictions to both approaches. In the first comparison, publicly available ESTs and mRNA transcripts were aligned to the human genome sequence. 13,934 exons with evidence for exon-skipping and/or inclusion (EST-SE for EST-verified skipped exons) were generated, comprising ∼7% of all internal exons. First we analyzed Cyt-ES versus hCNS-SCns. If we required that none of the points per probeset (exon) was significant, 6% (4,402 of 71,731) of exons (after probeset mapping) had evidence for EST-SE (Figure 6A). Shuffling the mapping between these probesets and exons resulted in 8% (5,777 of 71,731) of exons with evidence for EST-SE (Figure 6A). These percentages were not significantly different from the 7% of exons with EST evidence for AS observed from using all exons. By raising the requirement that probesets had to contain at least one significant point to five significant points, the percentage of EST-SE increased dramatically from 11% (531 of 4,898 exons) to 26% (33 of 126). In comparison, the shuffled probesets at the same requirements remained at ∼8%, rising slightly to 11% at five points, due to small sample sizes. Similar trends were observed with hCNS-SCns versus HUES6-ES and the derived NPs versus hESCs (Figure 6A). Therefore, we concluded that REAP[+] exons were enriched for AS events independently identified by a transcript-based approach.

Next, we compared REAP predictions to a computational approach of identifying exons with AS conserved in human and mouse, ACEScan [55]. ACEScan receives as input orthologous human–mouse exon pairs and flanking intronic regions and computes sequence features and integrates the features into a machine-learning algorithm to assign a real-valued score to the exon. A positive score indicated a higher likelihood of being AS in both human and mouse. ACEScan was updated in the following ways. Firstly, instead of relying on orthology information by Ensembl, and then aligning flanking introns in “orthologous” exons, conserved exonic and intronic regions in human and mouse from genome-wide multiple alignments were extracted. Secondly, whereas in our previous analysis exons from the longest transcript in Ensembl were utilized, now we collapsed all the transcripts available at the UCSC genome browser and analyzed all exons in the entire gene loci. ACEScan was utilized to assign ACEScan scores to all ∼162,000 internal exons in our genes. Exons annotated as first or last exons in Refseq mRNAs were excluded from our analysis, resulting in 4,487 positive-scoring exons, 2-fold more exons than originally published.

Here we repeated our analysis with exons with positive ACEScan scores (ACE[+]) instead of EST-SEs. If we required that none of the points per probeset (exon) was significant, 2% (1,645 of 71,731) of exons (after probeset mapping) were ACE[+] (Figure 6B). Shuffling the mapping between these probesets and exons resulted in 3% (2,044 of 71,731) of exons being ACE[+] (Figure 6B). These percentages were not significantly different from the 2.7% observed from all exons (4,487 of the 162,000 exons that were scored by ACEScan). By raising the requirement that probesets had to contain five significant points, the percentage of ACE[+] exons increased from 4% to 11%. However, the sample sizes were small. In comparison, the shuffled probesets at the same requirements remained at ∼4%. Similar overall trends were observed with hCNS-SCns versus HUES6-ES and the derived NPs versus hESCs (Figure 6B). In total, 7.5% (131 of 1,737) of REAP [+] exons were designated as ACEScan[+] compared to 2.4% (2,328 of 97,437) of REAP[−] exons. This result suggested that a small but significantly enriched fraction of AS events in hESCs versus NPs was likely to be evolutionarily conserved in human and mouse. In conclusion, our results suggested that REAP predictions were congruent with predictions from two independent, orthogonal methods.

### Experimental Validation of Alternative Exons

The sensitivity and specificity of REAP in the identification of REAP[+] exons was tested by RT-PCR. To validate REAP[+] alternative exons, RT-PCR primers were designed in the flanking exons to amplify both isoforms. To be a positively validated candidate, the PCR products on a gel had to satisfy all of the following criteria: (i) at least one isoform with the expected size must be visible in each cell type; (ii) the relative abundance of the two isoforms must be altered between two cell types and the direction of change have to be consistent with the REAP studentized residuals: in our study positive residuals implied inclusion in hESCs and skipping in NPs, and negative residuals implied inclusion in NP and skipping in hESCs; and (iii) the results were replicable in at least two experiments.

**Figure 7:**
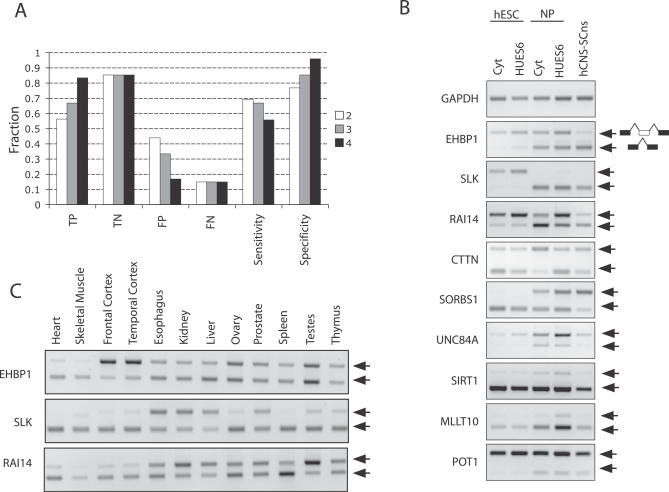
RT-PCR Validation of REAP-Predicted Alternative Exons (A) Probesets (exons) were considered REAP[+] candidates if they contained at least N = 2 (white bars), 3 (gray bars), or 4 (black bars) significant outliers. True positive (TP), true negative (TN), false positive (FP), and false negative (FN) rates were calculated from RT-PCR-validated REAP[+] exons at the different cutoffs (N = 2, 3, 4). (B) Nine RT-PCR validated REAP[+] AS events in hESCs (Cyt-ES and HUES6-ES), derived NPs (Cyt-NP and HUES6-NP), and hCNS-SCns. Arrows indicate the larger (exon-included) isoforms and smaller (exon-skipped) isoforms. (C) RT-PCR of REAP[+] alternative exons from *EHBP1*, *SLK*, and *RAI14* across a panel of human tissues. Arrows indicate the larger (exon-included) isoforms and smaller (exon-skipped) isoforms.

For simplicity of design, we tested candidates predicted from Cyt-ES versus hCNS-SCns. Fifteen REAP[+] exons with at least two significant outliers (out of five) were randomly chosen as predicted alternative events and thirty-five exons with less than two significant outliers were randomly chosen as constitutive events (Table S3). Nine of the fifteen exons (60%) were validated as AS events by our criteria. The sensitivity and specificity of the algorithm at the cutoff of two is 69% and 77%. Increasing the cutoff to three increased the specificity to 85%, with a slight decrease in sensitivity to 67% (Figure 7A). The patterns of AS in hESCs were similar in both Cyt-ES and HUES6-ES for all AS events validated, but the NPs (Cyt-NP, HUES6-NP, and hCNS-SCns) had more varied AS. The pattern of AS in the REAP[+] exons in the *SLK* (serine/threonine kinase 2) and *POT1* (protection of telomeres 1) genes showed remarkable agreement within derived NPs and hCNS-SCns (Figure 7B). The AS exon in *SLK* was observed to be included in hESCs and completely excluded in NPs; the AS exon in the *POT1* gene was included more in hESCs and a smaller isoform persisted in NPs. The AS patterns of the other verified REAP[+] exons were consistently similar in hESCs but were more varied in the NP. Interestingly, the patterns of AS in the derived NPs (Cyt-NP and HUES6-NP) were not always identical to those of hCNS-SCns. For example, the AS exon in the *EHBP1* (EH domain binding protein 1) gene was included in hESCs but skipped in hCNS-SCns, and both isoforms were present in the derived NPs (Figure 7B). As another example, the AS exon in the *SORBS1* (sorbin and SH3 domain containing 1) gene was skipped in hESCs and included in hCNS-SCns, but exhibited an intermediate pattern in the derived NPs. However, in some cases, the AS patterns in the derived NPs were different from both hESCs and hCNS-SCns (such as in the AS exon in UNC84A, *SIRT1*, and *MLLT10*).

First, given three independent samples each from two conditions, we concluded that REAP was able to identify AS events with high specificity but with moderate sensitivity. Second, AS events in hESCs were more similar, whereas the AS events in derived NPs were consistent with or intermediate to the benchmark hCNS-SCns, likely reflecting differences in the cell lines and/or differentiation protocols. In addition, we tested the AS patterns of REAP[+] exons from *EHBP1*, *SLK*, and *RAI14* in a panel of differentiated human tissues (Figure 7C). The REAP[+] alternative exon in the *RAI14* (retinoic acid induced 14) gene was observed to have the same AS pattern in NPs as in frontal and temporal cortex and in several other, non-brain adult tissues, such as heart and spleen. The AS pattern of the REAP[+] exon in the *SLK* gene in NPs was similar to most differentiated tissues; however, the relatively strong inclusion of the exon in hESCs was unique. Even in esophagus, kidney, liver, and prostate, both isoforms were present. The relative ratio of the exon-included to exon-skipped isoforms in SLK likely represents an ESC-specific AS signature. The alternative exon in the *EHBP1* gene was unusual. The exon was included in hESCs but also in frontal cortex and temporal cortex, a finding that was unexpected given the exclusion of the exon in hCNS-SCns (Figure 7C). The AS pattern in hCNS-SCns may represent a transient, early neuronal molecular change.

### Functional and Expression Characteristics of REAP[+] Genes

In total, 1,500 genes were identified that contained 1,737 REAP[+] exons, 68% of which lacked prior transcript (EST/cDNA) evidence for AS. To determine whether genes that contained REAP[+] exons, which we refer to as REAP[+] genes, are biased toward particular biological activities, REAP[+] genes were compared to a set of REAP analyzed genes not found to have REAP[+] exons (REAP[−] genes). A Gene Ontology analysis revealed that REAP[+] genes are enriched for GO molecular function categories “ATP binding,” “helicase activity,” “protein serine/theronine kinase activity,” “small GTPase regulatory/interacting protein activity,” and “thyroid hormone receptor binding” (Table 1). In terms of GO biological process categories, REAP[+] genes were more frequently involved in “ubiquitin cycle.” Similar results were obtained when we compared REAP[+] genes to all human genes that did not contain REAP[+] exons (Table 1) [55].

**Table 1:**
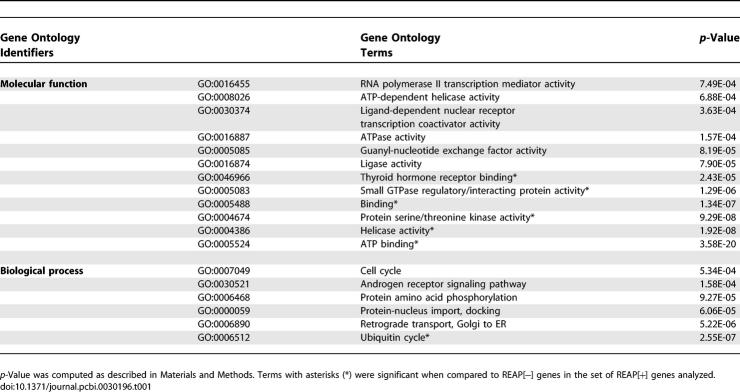
Significantly Enriched Gene Ontology Terms in REAP[+] Genes (Cutoff of Two Significant “Outliers” per Probeset)

**Figure 8:**
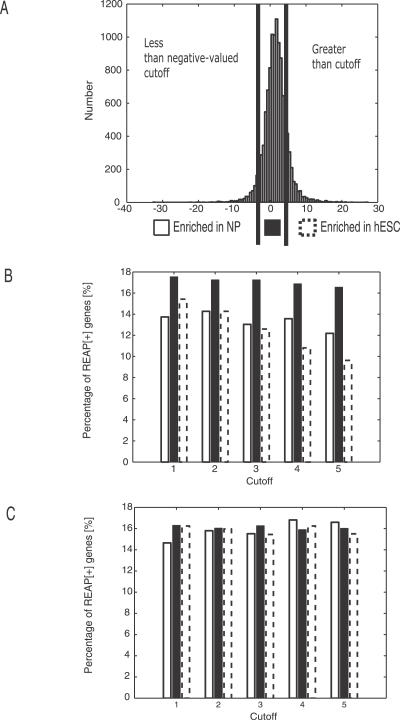
Analysis of REAP[+] Genes Relative to Transcriptional Differences (A) Histogam of *t*-statistics computed from gene-level signal estimates measuring the enrichment of genes in hESC and in NP. Genes on the right of the vertical line at 5 were designated enriched in hESC and genes on the left of the vertical line at −5 were designated enriched in NP; genes in between −5 and 5 were designated as “unchanged” or expressed similarly in hESC and NP. (B) Vertical bars representing the percentage of REAP[+] genes out of all genes in the different classifications (dashed bar: “enriched in hESC”; black filled bar: “unchanged”; white filled bar: “enriched in NP”), at different cutoffs of 1 to 5. (C) Set of genes where REAP[+] designation was randomly chosen. Similar representation as in (B).

Next we asked if REAP[+] genes are differentially expressed in hESCs compared to NPs and vice versa. For this analysis, the *t*-statistics computed above measuring the enrichment of a gene in hESCs relative to NPs was utilized for only REAP-analyzed genes. At a defined absolute-valued cutoff, genes were divided into three categories: “enriched in hESCs,” “enriched in NP,” or “unchanged” (Figure 8A). Increasing the *t*-statistic cutoff from one to five, the fraction of REAP[+] genes relative to REAP-analyzed genes remained constant in the “unchanged” categories (Figure 8B). However, the fraction of REAP[+] exons decreased significantly in “enriched in hESCs” and “enriched in NPs” categories. If we increased the cutoffs on genes that were randomly assigned as REAP[+] and REAP[−], controlling for the same number of genes in each category, we observed that the fraction of REAP[+] exons remained unchanged for all three categories (Figure 8C). To illustrate, at a cutoff of five, 10% (29 of 267) of enriched NP genes were REAP[+] genes and 8.8% (102 of 1,162) of enriched hESC genes were REAP[+], significantly different (*p* < 0.000005) from the random control where ∼14% of enriched NP and enriched hESC genes were REAP[+]. At a cutoff of five, 14% (1,368 of 9,636) of genes that were expressed at similar levels between hESCs and NPs were REAP[+]. Our results suggested that a strategy of focusing on differentially expressed genes would miss at least 14% of transcriptionally unchanged genes that may nevertheless have functional AS differences between hESCs and NPs.

### Conserved Intronic Splicing Regulatory Elements Proximal to REAP[+] hESC and NP Exons

Many, if not most, alternative exons undergo cell type–specific regulation by the binding of *trans*-factors to splicing regulatory *cis*-elements located proximal to or within the exons. As many tissue-specific splicing *cis*-regulatory elements were localized in intronic regions of AS exons, we focused on the identification of intronic splicing regulatory elements (ISREs) proximal to REAP[+] exons. In addition, we wanted to identify both common and cell type–specific ISREs. Three sets of exons were generated: (i) REAP[+] exons that were predicted to be included in NPs and skipped in hESCs (REAP[+]^NP^); (ii) REAP[+] exons that were predicted to be included in hESCs and skipped in NPs (REAP[+]^hESC^); and (iii) all REAP[−] exons. Regions of 400 base pairs flanking the exons were targeted for search. Initially, 5-mers that were significantly enriched between the upstream and downstream intronic regions of REAP[+]^NP^ and REAP[+]^ES^ relative to REAP[−] exons were enumerated. We were not able to identify 5-mers that were statistically significantly different.

**Figure 9:**
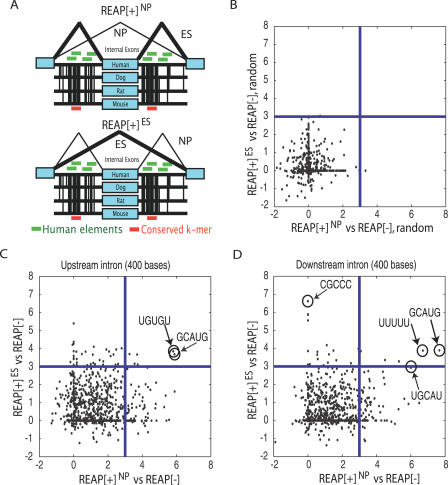
Conserved Intronic *cis-*Elements Enriched Proximal to REAP[+] Alternative Exons (A) Schematic describing the enumeration of intronic elements across 400 bases of flanking mammalian introns (human, dog, rat, and mouse). Red and green horizontal bars represent conserved intronic elements and nonconserved elements, respectively. Internal exons were divided into REAP[+]^NP^, REAP[+]^ES^, and REAP[−] exons. The χ statistic was computed to represent the enrichment of conserved elements in intronic regions flanking REAP[+]^NP^ versus REAP[−] exons (*x*-axis), and REAP[+]^ES^ versus REAP[−] exons (*y*-axis). The sign represented the direction of change, i.e., positive if enriched in introns flanking REAP[+] versus REAP[−] exon. Each conserved 5-mer was associated with two numbers: the enrichment in introns proximal to REAP[+]^NP^ versus REAP[−] exons (*x*-axis), and REAP[+]^ES^ versus REAP[−] exons (*y*-axis). (B) Downstream intronic regions, where the association between REAP[+] designation and the exons was shuffled. (C) Upstream intronic regions. Circled 5-mers in the upper right quadrant represent conserved 5-mers enriched in the upstream intronic regions of REAP[+]^NP^ and REAP[+]^ES^ exons. (D) Downstream intronic regions. Circled 5-mers in the upper right quadrant represent conserved 5-mers enriched in the downstream intronic regions of REAP[+]^NP^ and REAP[+]^ES^ exons.

Next, we focused on splicing signals that were conserved across mammalian genomes as a way of enhancing the signal of detecting functional splicing regulatory sequences [66]. Exons that were orthologous across human, dog, rat, and mouse were obtained and the flanking intronic regions were aligned (400 bases upstream and downstream separately; Figure 9A). We enumerated k-mers that were perfectly conserved across all four genomes in the upstream (and downstream) intronic regions. Each conserved k-mer was attributed a χ score representing its enrichment in a set of exons relative to another set of exons. The higher the score, the more frequent the conserved k-mer was in the first set relative to the second set. As a negative control, the associations between REAP scores and exons were shuffled. The enrichment scores for all downstream intronic 5-mers for shuffled REAP[+]^NP^ versus set REAP[−] exons (*x*-axis), and for shuffled REAP[+]^ES^ exons versus REAP[−] exons (*y*-axis) were displayed (Figure 9B). At a χ cutoff of three, which corresponded to a *p*-value of 0.0015, the majority of 5-mers were not significantly enriched in either shuffled set. Confident that no association of k-mers with shuffled REAP exons were found; we repeated the analyses for upstream and downstream intronic 5-mers for the original unshuffled sets. We identified 68 conserved 5-mers enriched upstream of REAP[+]^NP^ exons; and 34 5-mers enriched upstream of REAP[+]^ES^ exons (Figure 9C; Table S4). Of the 5-mers that were significantly enriched upstream of REAP[+]^NP^ exons, we identified a U-rich motif (UUUUU), a GU-rich motif (GUGUG), and a CU-rich motif (CCUCU, CUCUC, UCUCU, GCUCU). It is known that the heterogeneous ribonucleoprotein C (*hnRNP C*) binding site obtained by SELEX is five “U”s [67]. GU-rich sequences in flanking intronic regions were shown to bind to splicing factor *ETR-3* to regulate AS [68]. CU-rich sequences were shown to bind the splicing factor *PTB* [69]. Of the 5-mers enriched upstream of REAP[+]^ES^ exons, we observed CUAAC, which resembled the splicing branch-signal. Of the six 5-mers that were enriched upstream of both REAP[+]^NP^ and REAP[+]^ES^ exons, we identified GCAUG, which was previously shown to be an intronic splicing *cis*-element for the mammalian fibronectin and calcitonin/CGRP genes [70–72]. More recently, both mammalian *Fox1* and *2* have been demonstrated to regulate alternatively spliced exons via UGCAUG binding sites in neighboring introns in neuronal cell cultures [73].

Eighteen conserved 5-mers were significantly enriched in the downstream introns of REAP[+]^ES^ exons; and 76 5-mers were enriched downstream of REAP[+]^NP^ exons (Table S4, Figure 9D). We identified a motif CUCAU resembling the Nova binding site YCAY [74], and a G-rich motif (AGGGG, GGGGA, GGGGC, GGGGG, GGGGU) enriched in the introns downstream of REAP[+]^ES^ exons. G-rich motifs had previously been shown to be part of a bipartite signal that silences AS exons [75]. Of the five 5-mers that were enriched downstream of both REAP[+]^NP^ and REAP[+]^ES^ exons, GCAUG and a U-rich motif (UUUUU) were identified. We concluded that potential ISREs were enriched proximal to a subset of REAP[+] exons; in particular, the *Fox1/2* binding site GCUAG may play a regulatory role in controlling AS events in hESCs and NPs.

## Discussion

The ability of ESCs to generate all three embryonic germ layers has raised the exciting possibility that hESCs may become an unlimited source of cells for transplantation therapies involving organs or tissues such as the liver, pancreas, blood, and nervous system, and become tools to explore the molecular mechanisms of human development. Despite such interests, relatively little is understood about the molecular mechanisms defining their pluripotency and the molecular changes important for hESCs to differentiate into specific cell types. To understand these events, protocols are still being developed to differentiate ESCs into a variety of lineages.

Of particular biomedical interest is in the capacity of hESCs to be differentiated into a self-renewing population of NPs that can be then further coaxed into a variety of neuronal subtypes, such as dopaminergic neurons that are important in the treatment of Parkinson disease or cholinergic neurons for ALS (amyotrophic lateral sclerosis). While many microarray studies have explored molecular differences between hESCs and derived NPs, most, if not all, have focused on transcriptional changes. These studies have largely ignored intermediate RNA processing events prior to and during translation. In recent years, AS has gained momentum as being important in development, apoptosis, and cancer.

REAP, a regression-based method for analyzing exon array data was introduced, and was applied to discover AS events in hESCs, their derived NPs, and in hCNS-SCns. REAP was based on the assumptions that most exons in the gene of interest and in the genome are constitutively spliced and that outliers in a linear pairwise comparison of the signal estimates for probesets in a gene could be detected using a robust regression-based approach. REAP predictions were found to correlate well with transcript-based methods for identifying alternative exons, which interestingly suggested that current databases of transcript information, albeit not specifically enriched for hESC or NPs, in aggregate are nevertheless predictive of AS events in hESC and NP. In addition, REAP[+] exons were also enriched for ACEScan-predicted evolutionarily conserved exons [55]. As ACEScan utilized a different set of information from REAP, the agreement between both algorithms served to further validate the predicted alternative exons. Additional studies in mouse ESCs and neural derivatives will be necessary to determine if these AS events are indeed preserved in these analogous and orthologous cell types.

Our finding that only a minority of AS events was common between various hESC to NP comparisons is intriguing. A possible explanation is that the cell lines were not only genetically different, but were also exposed to different isolation and culture conditions. In addition, the different differentiation protocols established as optimal for generating *Nestin* and *Sox1* positive neural precursors may lead to vastly different molecular changes. It is likely that post-transcriptional changes such as AS may be more variable despite the cells being at acknowledged “end-points” defined by a limited set of immunohistochemical markers. Our results are consistent with a recent study that showed that while two well-established hESC lines differentiate into functional neurons, the two lines exhibited distinct differentiation potentials, suggesting that some preprogramming had occurred [76]. In particular, microRNA profiling revealed significant expression differences between the two hESC lines, suggesting that microRNAs, known post-transcriptional regulators, may sway the differentiation properties of the cell lines [76]. We postulated that AS events may serve also to bias the differentiation spectrum of the cells, an important avenue for future work.

Experimental validation of REAP[+] exons suggested a high specificity at the expense of relatively moderate sensitivity. We believe that the high FP rates may arise from cross-hybridization effects that remained unaccounted for. However, our specificity of 77% at the cutoff of two significant outliers per probeset allowed us to estimate that at least 1,336 of 1,737 REAP[+] exons were true AS events that changed during neuronal differentiation of hESC cells, and/or were different between endogeneous NPs and hESC. On average, 7% of all human exons have been estimated by transcript data to undergo AS; thus REAP's validation rate of 60% at the cutoff of two is 73-fold (60/7) higher than expected. In addition, we validated nine novel AS events that distinguish hESCs and NPs. Consistent with our computational results, we observed that the AS patterns in hCNS-SCns were not always similar to those of the derived NPs. It was important to point out that while transcriptional expression of these genes did not distinguish these cells from one another, in several instances the REAP-predicted AS event was able to separate derived NPs and hCNS-SCns. A notable exception was the alternative exon in the *SLK* gene, encoding a serine/threonine kinase protein, which was commonly included in both hESCs, i.e., the exon-excluded isoform was not present in hESCs compared to NPs, as well as in a variety of differentiated tissues. Closer inspection of the REAP[+]-validated AS exon in the SLK gene revealed strong conservation in the intronic region flanking the exon, a hallmark feature of evolutionarily conserved AS exons [55,77,78]. A study analyzing the expression patterns of the *SLK* gene suggested a potential functional role during embryonic development and in the adult central nervous system [79]; however, to our knowledge, our identification of the *SLK* alternative exon is the first report of a hESC-biased AS pattern during neuronal differentiation and across a myriad of differentiated tissues. In agreement, GO analysis suggested that genes containing REAP[+] exons were enriched in serine/threonine kinase activity, of which *SLK* is a family member. Future work will be required to study the impact of AS in these genes in hESCs and NPs. We predict it is unlikely that the alternative exon in the *SLK* gene is the only case common across hESC and different from differentiated tissues, but further studies will be necessary to identify other hESC-specific exons.

REAP[+] exons were underrepresented in genes that were differentially transcriptionally regulated in hESCs and NPs. Our results act as a reminder that focusing only on genes that are differentially expressed will overlook RNA processing events that may be biologically relevant to the system of interest. Finally, we identified potential *cis-*regulatory intronic elements conserved and enriched proximal to the REAP[+] exons. In particular, the *FOX1/2* binding site, GCUAG, was conserved and enriched in the flanking introns of a subset of REAP[+] exons. Further studies will be required to explore the importance of *FOX1* family members in early neuronal differentiation.

In conclusion, our introduction of REAP and its application to identifying AS events has revealed new and unanticipated insights into hESC biology and their transition to NP cells. Collectively, these exons represent a set of molecular changes that are likely to be important for studying human neural differentiation with applications in neuronal regenerative medicine.

## Materials and Methods

### Maintenance and differentiation of hESCs and hCNS-SCns.

hESC line Cy203 (Cythera) was cultured as previously described [12]. To differentiate into neuroepithelial precursor cells, colonies were manually isolated from mouse embryonic fibroblasts (MEFs) and cut in small pieces. These pieces were transferred to a T75 flask with hESCs differentiation media (same hESC medium but 10% KSR and no FGF-2). Medium was changed the next day by transferring the floating hESC aggregates to a new flask. After culturing for a week, the hESC cell aggregates formed mature embroid bodies (EBs; ∼10 um round clusters with dark centers). EBs were plated on a coated 10-cm dish in hESC differentiation media. The next day, the medium was changed to DMEM/F12 supplemented with ITS and fibronectin. Medium was changed every other day for a week or until the cells formed rosette-like columnar structures that were isolated manually. These structures were then transferred to coated dishes in neural induction medium (DMEM/F12 supplemented with N2 and FGF-2) for a week. Elongated single cells were separated from leftover aggregates using non-enzymatic dissociation. After one to two passages, the cells formed a monolayer of homogeneous NPs (negative for Sox1 immunostaining). Upon confluence, cells will form neurospheres that can also be isolated from the neuroepithelial precursor cells (positive for Sox1 immunostaining). At any of these two stages, pan-neuronal differentiation can be achieved after three to four weeks. hESC line HUES6 was cultured on MEF feeders as previously described (http://www.mcb.harvard.edu/melton/hues/) or on GFR matrigel coated plates. Cells grown on matrigel were grown in MEF-conditioned medium and FGF-2 was used at 20 ng/mL instead of 10 ng/mL for cells grown on MEFs. To differentiate neuroepithelial precursors, colonies were removed by treatment with collagenase IV (Sigma) and washed three times in growth media. The pieces of colonies were resuspended in HUES growth media without FGF2 in an uncoated bacterial Petri dish to form EBs. After one week, EBs were plated on polyornathine/laminin coated plates in DMEM/F12 supplemented with N2 and FGF2. Rosette structures were manually collected and enzymatically dissociated with TryPLE (Invitrogen), plated on polyornathine/laminin coated plates, and grown in DMEM/F12 supplemented with N2 and B27-RA and 20 ng/mL FGF-2. Cells could be grown as a monolayer for up to at least ten passages. Cells were Sox1 and nestin positive and readily differentiated into neurons upon withdrawal of FGF-2. Human central nervous system stem cell line FBR1664 (StemCells) which is referred to as hCNS-SCns in the main text was cultured as previously described [23]. The cells were cultured in medium consisting of Ex Vivo 15 (BioWhittaker) medium with N2 supplement (GIBCO), FGF2 (20 ng/mL), epidermal growth factor (20 ng/mL), lymphocyte inhibitory factor (10 ng/mL), 0.2 mg/ml heparin, and 60 ug/mL N-acetylcysteine. Cultures were fed weekly and passaged at ∼two to three weeks using collagenases (Roche). The following antibodies and corresponding dilutions were utilized for the immunohistochemical analysis of marker genes in Cyt-ES and HUES6-ES: Sox2 (Chemicon, 1:500), Oct4 (Santa Cruz, 1:500), Sox1 (Chemicon, 1:500), Nestin (Pharmingen, 1:250); hCNS-SCns: Sox2 (Chemicon, 1:200), Nestin (Chemicon, 1:200).

### RNA preparation and array hybridization.

Total RNA from cells was processed as follows. Cells were lysed in 1 mL of RNA-bee (Teltest). The RNA was isolated by chloroform extraction of the aqueous phase, followed by isopropanol precipitation as per the manufacturer's instructions. The precipitated RNA was washed in 75% ethanol and eluted with DEPC-treated water. Five ug of RNA was treated with RQ1 DNAase (Promega) according to the manufacturer's instructions. One ug of total RNA for each sample was processed using the Affymetrix GeneChip Whole Transcript Sense Target Labeling Assay (Affymetrix). Ribosomal RNA was reduced with the RiboMinus Kit (Invitrogen). Target material was prepared using commercially available Affymetrix GeneChip WT cDNA Synthesis Kit, WT cDNA Amplification Kit, and WT Terminal Labeling Kit (Affymetrix) as per manufacturer's instructions. Hybridization cocktails containing ∼5 ug of fragmented and labeled DNA target were prepared and applied to GeneChip Human Exon 1.0 ST arrays. Hybridization was performed for 16 hours using the Fluidics 450 station. Arrays were scanned using the Affymetrix 3000 7G scanner and GeneChip Operating Software version 1.4 to produce .CEL intensity files.

### Detection of AS by RT-PCR.

cDNAs were generated from total RNA with Superscript III reverse transcriptase (Invitrogen). PCR reactions were performed with primer pairs designed for AS targets (annealing at 58 °C and amplification for 30 or 35 cycles). PCR products were resolved on either 1.5% or 3% agarose gel in TBE. The Ethidium Bromide-stained gels were scanned with Typhoon 8600 scanner (Molecular Dynamics) for quantification. The number of true positives (TP; false negatives, FN) was computed as the number of REAP[+] (REAP[−]) exons that were validated by RT-PCR as AS. The number of true negatives (TN; or FPs) was computed as the number of REAP[−] (REAP[+]) exons that were validated by RT-PCR as constitutively spliced. The true (false) positive rate was computed as TP (FP) divided by the total number of REAP[+] exons in the experimentally validated set. The true (false) negative rate was computed as the TN (FN) divided by the total number of REAP[−] exons in the experimentally validated set. Sensitivity was computed as TP/(TP+FN) and specificity was computed as TN/(FP+TN).

### Sequence databases.

Genome sequences of human (hg17), dog (canFam1), rat (rn3), and mouse (mm5) were obtained from UCSC, as were the whole-genome *MULTIZ* alignments [80]. The lists of known human genes (knownGene containing 43,401 entries) and known isoforms (knownIsoforms containing 43,286 entries in 21,397 unique isoform clusters) with annotated exon alignments to human hg17 genomic sequence were processed as follows. Known genes that were mapped to different isoform clusters were discarded. All mRNAs aligned to hg17 that were greater than 300 bases long were clustered together with the known isoforms. Genes containing less than three exons were removed from further consideration. A total of 2.7 million spliced ESTs were mapped onto the 17,478 high-quality genes to infer AS. Exons with canonical splice signals (GT-AG, AT-AC, GC-AG) were retained, resulting in a total of 213,736 exons. Of these, 197,262 (92% of all exons) were constitutive exons, 13,934 exons (7%) had evidence of exon-skipping, 1,615 (1%) exons were mutually exclusive alternative events, 5,930 (3%) exons had alternative 3′ splice sites, 5,181 (2%) exons had alternative 5′ splice sites, and 175 (<1%) exons overlapped another exon, but did not fall into the above classifications. A total of 324,139 probesets from the Affymetrix Human Exon 1.0 ST array were mapped to 208,422 human exons, representing 17,431 genes. These probesets were used to derive gene and exon-level signal estimates from the CEL files. The four-way mammalian (four-mammal) whole-genome alignment (hg17, canFam1, mm5, rn3) was extracted from the eight-way vertebrate *MULTIZ* alignments (hg17, panTrol1, mm5, rn3, canFam1, galGal2, fr1, danRer1) obtained from the UCSC Genome Browser. Four-way mammal alignments were extracted for all internal exons, and 400 bases of flanking intronic sequence, resulting in a total of 161,731 conserved internal exons. A total of 145,613 (90% of total) conserved internal exons were constitutive exons, 13,653 exons (8%) had evidence of exon-skipping, 1,576 exons were mutually exclusive alternative events, 5,818 exons had alternative 3′ splice sites, 5,046 exons had alternative 5′ splice sites, and 168 exons overlapped another exon.

### Exon array analysis.

The Affymetrix Power Tools (APT) suite of programs was obtained from http://www.affymetrix.com/support/developer/powertools/index.affx. Exon (probeset) and gene-level signal estimates were derived from the CEL files by RMA–sketch normalization as a method in the apt-probeset-summarize program. To determine if the signal intensity for a given probeset is above the expected level of background noise, we utilized the DABG (detection above background) quantification method available in the apt-probeset-summarize program as part of Affymetrix Power Tools (APT). Briefly, DABG compared the signal for each probe to a background distribution of signals from anti-genomic probes with the same GC content. The DABG algorithm generated a *p*-value representing the probability that the signal intensity of a given probe was part of the background distribution. We considered a probeset with a DABG *p*-value lower than 0.05 as detected above background. The statistic t_hCNS-SCns,ESC_ = (μ_hCNS-SCns_ − μ_ESC_) / sqrt (((n_hCNS-SCns_ − 1)σ^2^_hCNS-SCns_ + (n_ESC_ − 1)σ^2^_ESC_)(n_hCNS-SCns_ + n_ESC_)) / ((n_hCNS-SCns_n_ESC_) (n_hCNS-SCns_ + n_ESC_ − 2))), where n_hCNS-SCns_ and n_ESC_ were the number of replicates, μ_hCNS-SCns_ and μ_ESC_ were the mean, and σ^2^_hCNS-SCns_ and σ^2^_ESC_ were the variances of the expression values for the two datasets used to represent the differential enrichment of a gene using gene-level estimates in hCNS-SCns relative to hESCs. Multiple hypothesis testing was corrected by controlling for the false discovery rate (Benjamini-Hochberg).

### AS detection by REAP.

The log_2_ signal estimate x_ij_ for probeset i in cell-type j had to satisfy two conditions, otherwise the probeset was discarded: (i) 2 < x_ij_ < 10,000 for all conditions/cell-types j; and (ii) DABG *p*-value < 0.05 for all replicates in at least one condition/cell-type j. A gene had to have five probesets that satisfied the two conditions above in order to be considered for robust regression analysis. After generating the points (as described in the Results section), we utilized the robust regression method rlm in R-package “MASS” (version 6.1–2) with M-estimation and a maximum iteration setting of 30 to estimate the linear function y_i_ = αx_i_ + β. For each probeset, we computed the error term e_i,_, which was the difference between the actual value y_i_ and the estimated value ξ_i_, from the estimated function ξ_i_ = Ax_i_ + B, where A and B were estimates of α and β. The error term variance was estimated by s_e_^2^ = Σe_i_^2^/(n − p), which was used to estimate the variance of the predicted value, s_ξi_^2^ = s_e_^2^(n^−1^ + (x_i_ − μ_x_)^2^ / s_x_^2^(n − 1)). Here, n referred to the number of points (generated for each gene), and p referred to the number of independent variables (p = 2 in our method); and μ_x_ = Σx_i_^2^/n; s_x_^2^ = n^−1^ Σ(x_i_ − μ_x_)^2^. Following Belsley et al. [81], we defined the leverage h_i_ of the i^th^ point as h_i_ = n^−1^ + (x_i_ − μ_x_)^2^ / s_x_^2^(n − 1). Here we considered a point to have high leverage if h_i_ > 3p/n. Next, we calculated the covariance ratio, cov_i_ = (s_i_^2^/s_r_^2^)^p^/(1 − h_i_), which is the ratio of the determinant of the covariance matrix after deleting the i^th^ observation to the determinant of the covariance matrix with the entire sample. We considered a point to have high influence if |cov_i_ − 1| > 3p/n. Lastly, we computed the studentized residuals, rstudent_i_ = e_i_ / (s_(i)_^2^ (1 − h_i_)^0.5^), where s_(i)_^2^ = (n-p)s_e_^2^ / (n-p-1) – e_i_^2^ / (n-p-1)(1 − h_i_), the error term variance after deleting the i^th^ point. As rstudent_i_ was distributed as Student's *t*-distribution with n-p-1 degrees of freedom, each rstudent_i_ value was associated with a *p*-value. We considered a point to be an “outlier” if *p* < 0.01.

### Identification of motifs.

The enrichment score of a sequence element of length *k* (k-mer) in one set of sequences (set 1) versus another set of sequences (set 2) was represented by the nonparametric χ^2^ statistic with Yates correction, computed from the two by two contingency table, T (T_11_: number of occurrences of the element in set 1; T_12_: number of occurrences of all other elements of similar length in set 1; T_21_: number of occurrences of element in set 2; T_22_: number of occurrences of all other elements of similar length in set 2. All elements had to be greater than 5. To correct for multiple hypothesis testing, *p*-values were multiplied by the total number of comparisons.

## Supporting Information

Figure S1 Number of Overlapping and Non-Overlapping Transcriptionally Enriched Genes in NP and hESC between Pairwise Gene-Level Comparisons of Different hESC to NP Samples Displayed as Venn Diagrams For each hESC to NP pair, the percentage of enriched genes found in the intersection was indicated in parentheses.
(174 KB PDF)

Table S1 Number of Probesets with Significant “Outliers” (39 KB DOC)

Table S2 REAP[+] Exons Were Defined as Probesets Matching Internal Exons with at Least Two (or Three) Significant Points “1” in the table indicated that the event had to be present in the comparisons above (Cyt-ES versus hCNS-SCns; HUES6-ES versus hCNS-SCns; Cyt-NP versus Cyt-ES; HUES6-NP versus HUES6-ES).
(44 KB DOC)

Table S3 Experimental Validation of REAP[+] Targets “N” and “P” indicated negative or positive validation by RT-PCR. The genomic coordinates of the exon for hg17 were represented as chromosome, followed by start and end, separated by “:”. “1” and “−1” indicated whether the exon was REAP[+] “1” or REAP[−] “−1”, at the different cutoffs of one to four.
(130 KB DOC)

Table S4 Conserved 5-mers Enriched in Downstream/Upstream Intronic Regions of REAP[+] Exons Included in ES (NP) and Skipped in NP (ES) For example, in row 6 ACCTG was enriched in the downstream intronic regions of exons included in ES and skipped in NP, relative to REAP[−] exons.
(34 KB DOC)

## Abbreviations

AS: alternative splicing
DABG: detection above background
EB: embryoid body
EST: expressed sequence tag
hCNS-SCns: human central nervous system stem cells grown as neurospheres
hESC: human embryonic stem cell
NP: neural progenitor
NSC: neural stem cell
REAP: Regression-based Exon Array Protocol
