# Supplementary figures and images for: Alternative Splicing Events Identified in Human Embryonic Stem Cells and Neural Progenitors
Source: PLoS Comput Biol. 2007 Oct 26;3(10):e196. doi: 10.1371/journal.pcbi.0030196 (PMC2041973; doi:10.1371/journal.pcbi.0030196)

SUPPLEMENTARY FIGURE 1

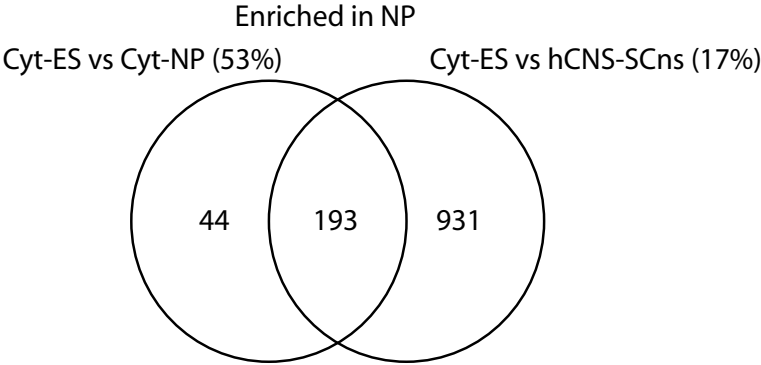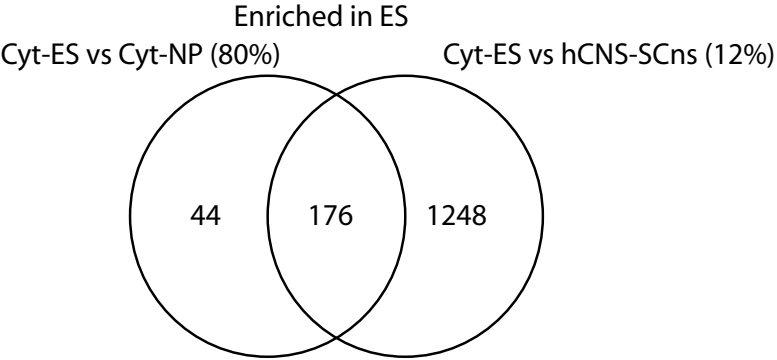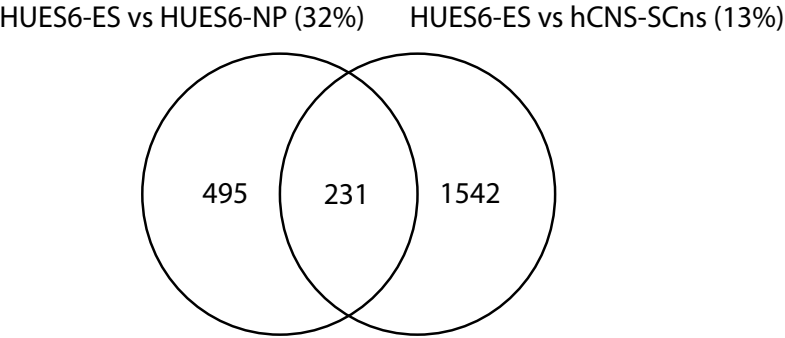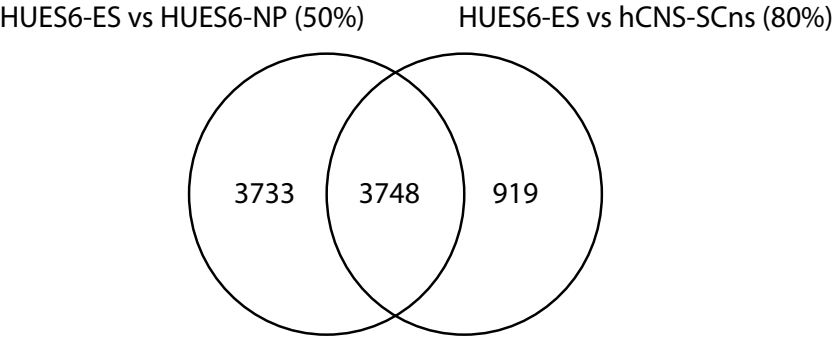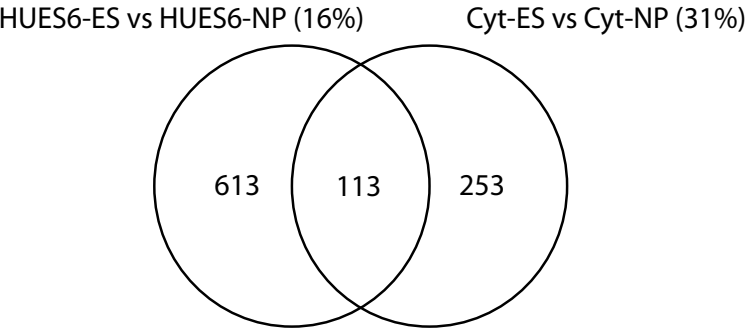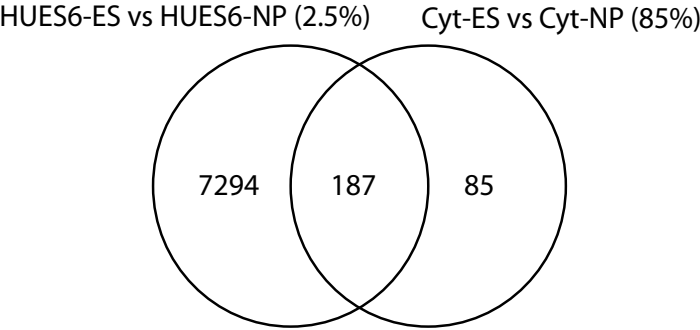

Supplement: Figure S1 — For each hESC to NP pair, the percentage of enriched genes found in the intersection was indicated in parentheses. (174 KB PDF) [file pcbi.0030196.sg001.pdf]
