# Supplementary material for: Alternative Splicing Events Identified in Human Embryonic Stem Cells and Neural Progenitors
Source: PLoS Comput Biol. 2007 Oct 26;3(10):e196. doi: 10.1371/journal.pcbi.0030196 (PMC2041973; doi:10.1371/journal.pcbi.0030196)
Supplement: Table S1 — (39 KB DOC) [file pcbi.0030196.st001.doc]

Supplementary Table 1. Number of probesets with significant ‘outliers’

| Number of significant points per probeset | (A) hCNS-SCns versus Cyt-ES | (B)  hCNS-SCns versus HUES6-ES | (C) Cyt-NPs versus Cyt-ES | (D) HUES6-NP versus HUES6-ES | (A) and (B) | (C) and (D) | (A), (B), (C), (D) |
| --- | --- | --- | --- | --- | --- | --- | --- |
| 0 | 109940 | 126310 | 117394 | 115780 | 139522 | 148615 | 158089 |
| 1 | 12566 | 10374 | 12927 | 5645 | 3539 | 1498 | 506 |
| 2 | 1192 | 1884 | 1567 | 1382 | 361 | 161 | 43 |
| 3 | 490 | 876 | 419 | 861 | 170 | 42 | 11 |
| 4 | 308 | 610 | 176 | 840 | 108 | 26 | 8 |
| 5 | 108 | 409 | 45 | 732 | 33 | 7 | 0 |
