# Supplementary material for: Alternative Splicing Events Identified in Human Embryonic Stem Cells and Neural Progenitors
Source: PLoS Comput Biol. 2007 Oct 26;3(10):e196. doi: 10.1371/journal.pcbi.0030196 (PMC2041973; doi:10.1371/journal.pcbi.0030196)
Supplement: Table S2 — “1” in the table indicated that the event had to be present in the comparisons above (Cyt-ES versus hCNS-SCns; HUES6-ES versus hCNS-SCns; Cyt-NP versus Cyt-ES; HUES6-NP versus HUES6-ES). (44 KB DOC) [file pcbi.0030196.st002.doc]

Supplementary Table 2. REAP[+] exons were defined as probesets matching internal

exons with at least two (or three) significant points. ‘1’ in the table indicated that the event had to be present in the comparisons above (Cyt-ES vs hCNS-SCns; HUES6-ES vs hCNS-SCns; Cyt-NP vs Cyt-ES; HUES6-NP vs HUES6-ES).

| hCNS-SCns vs Cyt-ES | hCNS-SCns vs HUES6-ES | Cyt-NP vs Cyt-ES | HUES6-NP vs HUES6-ES | Number of REAP[+] above 2 (3) |
| --- | --- | --- | --- | --- |
| -1 | 1 | -1 | -1 | 614 (281) |
| -1 | -1 | -1 | 1 | 439 (262) |
| -1 | -1 | 1 | -1 | 250 (42) |
| 1 | -1 | -1 | -1 | 220 (65) |
| 1 | 1 | -1 | -1 | 82 (41) |
| -1 | 1 | -1 | 1 | 44 (17) |
| 1 | -1 | 1 | -1 | 41 (9) |
| 1 | 1 | 1 | -1 | 12 (5) |
| -1 | 1 | 1 | -1 | 11 (2) |
| -1 | -1 | 1 | 1 | 8 (2) |
| 1 | -1 | -1 | 1 | 5 (3) |
| 1 | 1 | -1 | 1 | 5 (4) |
| 1 | 1 | 1 | 1 | 3 (0) |
| 1 | -1 | 1 | 1 | 2 (0) |
| -1 | 1 | 1 | 1 | 1( 0) |
|  |  |  | Total | 1,737 (733) |
