# Supplementary material for: Alternative Splicing Events Identified in Human Embryonic Stem Cells and Neural Progenitors
Source: PLoS Comput Biol. 2007 Oct 26;3(10):e196. doi: 10.1371/journal.pcbi.0030196 (PMC2041973; doi:10.1371/journal.pcbi.0030196)
Supplement: Table S3 — “N” and “P” indicated negative or positive validation by RT-PCR. The genomic coordinates of the exon for hg17 were represented as chromosome, followed by start and end, separated by “:”. “1” and “−1” indicated whether the exon was REAP[+] “1” or REAP[−] “−1”, at the different cutoffs of one to four. (130 KB DOC) [file pcbi.0030196.st003.doc]

Supplementary Table 3. Experimental validation of REAP[+] targets. ‘N’ and ‘P’ indicated negative or positive validation by RT-PCR. The genomic coordinates of the exon for hg17 were represented as chromosome, followed by start and end, separated by “:”. “1” and “-1” indicated whether the exon was REAP[+] “1” or REAP[-] “-1”, at the different cutoffs of one to four.

|  |  |  | Number of significant outliers per probeset | | | |
| --- | --- | --- | --- | --- | --- | --- |
| RT-PCR validation | Genome coordinates (hg17) | Gene description | 1 | 2 | 3 | 4 |
| N | 3:180577531-180577658 | mitofusin 1 isoform 2 | 1 | 1 | -1 | -1 |
| N | 10:90758625-90758734 | tumor necrosis factor receptor superfamily | -1 | -1 | -1 | -1 |
| N | 13:38348158-38348286 | FRAS1 related extracellular matrix protein 2 | -1 | -1 | -1 | -1 |
| N | 2:150258458-150258564 | chromosome 2 open reading frame 25 | 1 | 1 | -1 | -1 |
| N | 3:185552932-185552986 | chloride channel 2 | -1 | -1 | -1 | -1 |
| N | 10:134916356-134916507 | kinase non-catalytic C-lobe domain (KIND) | -1 | -1 | -1 | -1 |
| N | 12:51961557-51961677 | extra spindle poles like 1 | -1 | -1 | -1 | -1 |
| N | 11:6592977-6593136 | tripeptidyl-peptidase I precursor | -1 | -1 | -1 | -1 |
| N | 14:88726480-88726546 | checkpoint suppressor 1 | -1 | -1 | -1 | -1 |
| N | 11:10782259-10782326 | eukaryotic translation initiation factor 4 | 1 | -1 | -1 | -1 |
| N | 18:52505083-52505203 | WD repeat domain 7 protein isoform 1 | -1 | -1 | -1 | -1 |
| N | 16:88385835-88385977 | Fanconi anemia, complementation group A | 1 | 1 | 1 | 1 |
| N | 11:62289387-62289453 | DNA directed RNA polymerase II polypeptide G | -1 | -1 | -1 | -1 |
| N | 14:71241190-71241253 | signal-induced proliferation-associated 1 like | 1 | 1 | -1 | -1 |
| N | 15:20391684-20391781 | tubulin, gamma complex associated protein 5 | 1 | 1 | 1 | -1 |
| N | 14:91013005-91013083 | KIAA2010 isoform 2 | 1 | -1 | -1 | -1 |
| N | 14:91551792-91551958 | thyroid hormone receptor interactor 11 | 1 | -1 | -1 | -1 |
| N | 20:25207692-25207785 | brain glycogen phosphorylase | -1 | -1 | -1 | -1 |
| N | X:135038517-135038645 | Hypothetical protein FLJ12649 | -1 | -1 | -1 | -1 |
| N | 16:87307220-87307356 | hypothetical protein LOC348180 isoform 2 | -1 | -1 | -1 | -1 |
| N | 14:57895602-57895687 | retinoblastoma-binding protein 1 isoform I | -1 | -1 | -1 | -1 |
| N | X:48076357-48076437 | amino acid transport system N2 | 1 | -1 | -1 | -1 |
| N | 21:43920580-43920645 | hypothetical protein LOC23076 | 1 | 1 | 1 | -1 |
| N | 16:87221828-87222028 | hypothetical protein BC001584 | 1 | -1 | -1 | -1 |
| N | 8:99097943-99098066 | matrilin 2 precursor | -1 | -1 | -1 | -1 |
| N | 16:29925994-29926121 | double C2-like domains, alpha | -1 | -1 | -1 | -1 |
| N | X:135048838-135048956 | Hypothetical protein FLJ12649 | -1 | -1 | -1 | -1 |
| N | 14:67311485-67311584 | zinc finger, FYVE domain containing 26 | -1 | -1 | -1 | -1 |
| N | X:83167517-83167640 | ribosomal protein S6 kinase, 90kDa, polypeptide | -1 | -1 | -1 | -1 |
| N | 3:52158871-52159043 | DKFZP434C245 protein | -1 | -1 | -1 | -1 |
| N | 14:72648612-72648711 | RNA binding motif protein 25 | -1 | -1 | -1 | -1 |
| N | 6:33762163-33762274 | inositol 1,4,5-triphosphate receptor, type 3 | -1 | -1 | -1 | -1 |
| N | 10:34665132-34665177 | partitioning-defective protein 3 homolog | 1 | 1 | 1 | -1 |
| N | 7:76892447-76892533 | protein tyrosine phosphatase, non-receptor type | 1 | -1 | -1 | -1 |
| N | 11:102580760-102580903 | Hypothetical protein FLJ16607 | -1 | -1 | -1 | -1 |
| N | 4:186021259-186021365 | MLF1 interacting protein | -1 | -1 | -1 | -1 |
| P | 5:34849435-34849522 | retinoic acid induced 14 | 1 | 1 | 1 | -1 |
| P | 10:69335925-69336050 | sirtuin 1 | 1 | 1 | 1 | 1 |
| P | 7:102561729-102561776 | zuotin related factor 1 | 1 | -1 | -1 | -1 |
| P | 17:55306681-55306846 | delta-tubulin | -1 | -1 | -1 | -1 |
| P | 10:105760563-105760656 | serine/threonine kinase 2 | 1 | 1 | -1 | -1 |
| P | 2:44112939-44113085 | leucine-rich PPR motif-containing protein | 1 | 1 | 1 | 1 |
| P | 9:111904771-111904868 | sushi domain containing 1 | -1 | -1 | -1 | -1 |
| P | 7:91723056-91723178 | ocular development-associated gene | 1 | 1 | 1 | -1 |
| P | 10:21900909-21900961 | myeloid/lymphoid or mixed-lineage leukemia | 1 | 1 | 1 | 1 |
| P | 11:69945223-69945334 | cortactin isoform b | 1 | 1 | 1 | 1 |
| P | 6:53486960-53487094 | glutamate-cysteine ligase, catalytic subunit | -1 | -1 | -1 | -1 |
| P | 10:97184368-97184464 | sorbin and SH3 domain containing 1 | 1 | 1 | 1 | 1 |
| P | 2:63126716-63126824 | EH domain binding protein 1 | 1 | 1 | 1 | -1 |
| P | 10:103543659-103543745 | meningioma expressed antigen 5 (hyaluronidase) | -1 | -1 | -1 | -1 |
|  |  | TP | 0.435 | 0.562 | 0.667 | 0.833 |
|  |  | TN | 0.852 | 0.852 | 0.852 | 0.852 |
|  |  | FP | 0.565 | 0.438 | 0.333 | 0.167 |
|  |  | FN | 0.148 | 0.148 | 0.148 | 0.148 |
|  |  | SENS | 0.714 | 0.692 | 0.667 | 0.556 |
|  |  | SPEC | 0.639 | 0.767 | 0.852 | 0.958 |
